# Barriers to mental healthcare and treatment for people living with HIV in the Asia‐Pacific

**DOI:** 10.1002/jia2.25189

**Published:** 2018-10-05

**Authors:** Annette H Sohn, Jeremy Ross, Milton L Wainberg

**Affiliations:** ^1^ TREAT Asia, amfAR – The Foundation for AIDS Research Bangkok Thailand; ^2^ Department of Psychiatry Columbia University New York NY USA

**Keywords:** HIV, mental health, Asia, Pacific, treatment, research

Mental illness is a leading cause of disability in South East Asia among those 15 to 49 years of age, with over 7.6 million disability‐adjusted life‐years lost in 2016 alone 1. Regional studies have found that as much as 40% of adults attending outpatient HIV clinics in Asia‐Pacific countries have depression 2, 3. Concomitant mental illness is associated with late antiretroviral therapy (ART) initiation and lack of timely viral suppression in people living with HIV (PLHIV) 4, 5.

However, integration of mental health and HIV care is uncommon in the region. In a survey of global IeDEA (International epidemiology Databases to Evaluate AIDS) clinical sites, only 43% of those in the Asia‐Pacific reported screening for depression, 39% for substance use disorders, 11% for post‐traumatic stress disorder and 29% for any other mental disorder – overall, representing the *lowest levels* of mental health assessments across this global consortium 6. Unfortunately, this is not surprising in the light of data from the World Health Organization's (WHO) Mental Health Atlas that shows there are 2.5 mental health workers per 100,000 people in the South East Asia region and 10 per 100,000 in the Western Pacific region, which compares to 50 per 100,000 in the European region 7. There are three key barriers to increasing the pool of providers who can screen, diagnose and treat mental illness among Asia‐Pacific PLHIV.

*Barrier #1: Multiple stigmas*. With 5.2 million PLHIV, the region has the second largest epidemic in the world which is primarily concentrated among key populations, including men who have sex with men, people who inject drugs, sex workers and transgender individuals 8. HIV‐related stigma, drug‐use stigma and sexual minorities’ stigma (e.g. homophobia, transphobia) in healthcare settings pose major barriers for optimal HIV prevention and care. Stigma associated with mental illness has been widespread in Asia and is associated with negative impacts on health‐seeking behaviour. When combined, these multiple stigmas also impact the numbers of providers willing to provide mental healthcare to PLHIV in settings where key populations may also be criminalized 9, 10.
*Barrier #2: Lack of integration between HIV and mental healthcare*. Despite the prevalence of mental illness among PLHIV, mental health screening is not commonly integrated into HIV clinic procedures, which is problematic because the bulk of care to PLHIV in the region is delivered by HIV providers primarily focused on ART management. Public health systems have separately functioning HIV and mental health departments or ministries, and national policies and practices have rarely supported cross‐disciplinary collaboration or clinical management.
*Barrier #3: Limited data to drive policy change*. There is a relatively small evidence base around mental illness among PLHIV in the Asia‐Pacific that is primarily focused on depression as an outcome. There are fewer studies that evaluate the utility of interventions to improve mental healthcare delivery in the context of HIV care.

A key intervention to overcome the substantial human resource limitations for mental healthcare for PLHIV in the region is to train non‐mental health specialists to deliver basic psychotherapeutic interventions with expert supervision. An example of this is the WHO's Mental Health Gap Action Programme (mhGAP), which has developed tools to accelerate task‐shifting in low‐ and middle‐income country settings (LMICs) 11. This includes training to screen, diagnose and treat common mental illnesses through a standardized platform. While there are efforts to implement mhGAP in primary care settings in the region, including in Cambodia, Malaysia and the Philippines, the tools are rarely used in HIV care. There is substantial potential to improve access to mental healthcare for PLHIV by expanding the use of these interventions.

In addition, research should be conducted within real‐world care settings to determine optimal strategies to diagnose and treat those in need as well as quantify the impact on adherence, retention and mortality for mental healthcare interventions. Identification and evaluation of optimal implementation strategies to scale and sustain integrated HIV and mental healthcare could provide the data needed to justify proactive policies and the allocation of limited health resources. Implementation science offers a platform particularly well‐suited to conduct research around the intersection of HIV and mental health, and can address complex questions related to the adoption, adaptation, integration, scale‐up and sustainability of evidence‐based practices, as well as monitoring and evaluating of outcomes at the patient, provider and system levels. Evidence‐based tools and treatments developed in high‐income countries must be “translated” across cultures, which requires more than linguistic adaptation in order to balance fidelity (to the original intervention) and fit (for a novel context) to achieve the desired outcomes of the interventions 12, 13. Mental health implementation science acknowledges variations in local knowledge, norms, attitudes and beliefs about mental illness, including stigma, which would be particularly useful in the Asia‐Pacific region where there is extensive cultural, religious and linguistic variation.

Central to these potential solutions for bridging the gaps between HIV and mental health is public health leadership and the political will behind it to prioritize mental healthcare within HIV programmes. As external HIV funding declines to Asia‐Pacific LMICs 8, national programmes are increasingly focused on achieving the UNAIDS 90‐90‐90 targets. However, with only 53% treatment coverage in the region, national programmes already face major challenges in scaling up. Calls for more comprehensive care for non‐communicable diseases will not be met without stronger advocacy and domestic and international donor support for mental healthcare.

While countries in the Asia‐Pacific continue to make progress with HIV testing and treatment scale‐up, we need a clear way forward to address mental health challenges among PLHIV. Access to potent antiretroviral medicines and laboratory monitoring alone will not solve these problems, which pose very real threats to adherence, retention and quality of life. Delivering sustainable and cost‐effective mental health services and addressing human resource, data and policy gaps are essential to address the intersection between HIV and mental illness, and will help maintain the public health gains made in controlling regional HIV epidemics.

## Competing interests

AHS has received travel and research support to her institution from ViiV Healthcare.

## Authors’ contributions

AHS and MLW developed the idea for and drafted the Viewpoint. All authors participated in research think tanks where ideas presented in the Viewpoint were discussed, and revised and approved the text.
